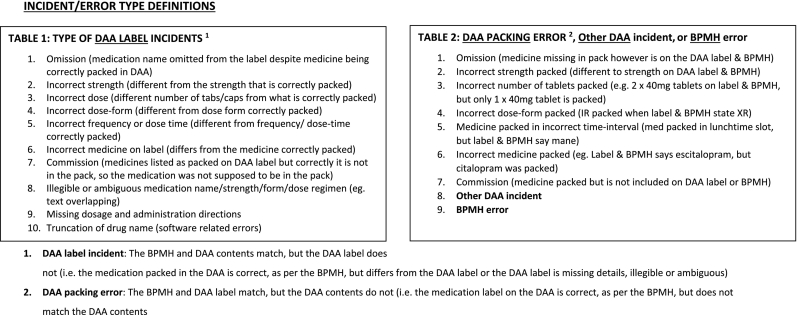# Accuracy of medication labels on community pharmacy-prepared dose administration aids: An observational study

**DOI:** 10.1016/j.rcsop.2023.100318

**Published:** 2023-08-11

**Authors:** Zulal Uzunbay, Rohan A. Elliott, Simone Taylor, Daniela Sepe, Emily J. Ferraro

**Affiliations:** a Pharmacy Department, Austin Health, Studley Rd, Heidelberg, Victoria 3084, Australia; b Faculty of Pharmacy and Pharmaceutical Sciences, Monash University, Parkville, Victoria 3052, Australia

## Abstract

**Background:**

Hospital prescribers often use the labels on multicompartment compliance aids or monitored dosage systems, known in Australia as dose administration aids (DAAs), as a trusted source of information about patients' medication regimens taken in the community.

**Aim:**

The primary aim was to explore the prevalence and nature of labelling incidents on community pharmacy-prepared DAAs.

**Methods:**

A convenience sample of 100 adult patients admitted to a metropolitan teaching hospital who used a community pharmacy-prepared DAA at home was recruited. Patients were excluded if their DAAs were not brought to hospital. As part of usual care, a pharmacist took a best possible medication history (BPMH) using multiple information sources. This ‘gold standard’ BPMH was compared to the regimen listed on the DAA summary label and the DAA contents. The primary outcome was the percentage of patients whose DAA summary label(s) had one or more incidents for DAA packed medications. DAA label incident was defined as incorrect, missing or illegible/ambiguous medication name, strength, dose or dose-form when compared to the BPMH and DAA contents. Secondary outcomes were compliance with best-practice guidelines for labelling DAAs; and percentage of patients with a DAA packing error.

**Results:**

The 100 patients used 110 DAAs, packed by 75 community pharmacies. Four (4.0%) patients had no medication summary label on their DAAs. Of the 96 patients whose DAA(s) had a summary label, 82 (85.4%) had one or more summary label incidents. The most prevalent incidents were ‘illegible, ambiguous or missing medication details’, ‘truncated medication name’ and ‘omission of a medication’. The most prevalent guideline non-compliance was not including generic medication names (68% DAA-packed medications). Two DAA packing errors were identified.

**Conclusion:**

A high prevalence of DAA labelling incidents was identified. Improved DAA labelling software functionality, more robust pharmacy procedures and pharmacy staff education are required.

## Keywords/MeSH terms

Drug packaging

Medication adherence

Medication errors

Pharmacists

Continuity of patient care

## 1 Introduction

Dose administration aids (DAAs), also known as ‘multicompartment compliance aids’ or ‘monitored dosage systems’, are medication management systems that allow medications to be repackaged and organised according to the prescribed dosing schedule.^1^, ^2^, ^3^ The aims of DAAs are to simplify medication-taking and improve medication adherence.^2^, ^3^, ^4^, ^5^

There are two types of sealed DAAs provided by community pharmacies in Australia: blister packs (e.g. Webster Pak, Medico Pak) and sachet systems (a continuous roll of individually labelled sachets each containing medications for one dose-time).^3^ A 2016 survey of a representative sample of Australians aged 50 years and over and using medications for at least one chronic condition reported that 16% used pharmacy filled DAAs.^6^

The Pharmaceutical Society of Australia has produced best-practice guidelines that outline labelling and packaging requirements for pharmacists providing DAA services.^7^ Pharmacies providing DAA packing services must ensure devices are clearly and accurately labelled with patient and medication details. One reason why it is important that DAA labels accurately reflect the contents of the DAA is because when a patient presents to hospital, the admitting doctor will often use the DAA label to chart the patient's medications, on the assumption that the DAA label is an accurate reflection of the patient's current medication regimen. Often it is not possible to verify the accuracy of the label with the patient, for example because people who use DAAs commonly have poor knowledge of their medication regimen.^3^^,^^8^ Prescribing errors may occur when admitting doctors rely on a DAA label to chart medications in hospital.

There has been limited research evaluating the accuracy and clarity of pharmacy-prepared DAA labels,^9^, ^10^, ^11^ and no previous study has compared DAA labels against professional practice guidelines for pharmacist DAA packing services. The primary aim of this study was to determine the prevalence and nature of DAA labelling incidents in patients presenting to hospital with a pharmacy-prepared DAA. Secondary aims were to assess compliance with Australian best-practice guidelines for DAA labelling, and determine the prevalence of DAA packing errors.

## 2 Method

This prospective observational study was undertaken at a large metropolitan public teaching hospital in Melbourne, Australia. A convenience sample of 100 patients who were using a community pharmacy-prepared DAA prior to hospital admission was recruited over three time periods as part of intern pharmacist research projects: August to November 2018 (40 patients), June to October 2019 (35 patients) and March to August 2020 (25 patients). The study was approved by the institution's Office for Research as an audit activity and individual patient consent was not required. This study is reported in accordance with the STROBE checklist for observational studies.^12^

Patients using a pharmacy-prepared DAA in the community were identified by searching the ‘patient's own medicines’ tubs stored in the hospital ward medication rooms, and from referrals from ward pharmacists. Patients were excluded if they (or a carer) prepared their DAA or their DAA was not brought to hospital. Patients using multiple DAAs concurrently (e.g. two blister pack DAAs) were excluded if all DAAs were not brought into hospital. Patients were also excluded if they did not have a pharmacist-prepared medication history against which the DAA label and contents could be compared.

### 2.1 Data collection tool

A data collection tool was created and piloted by the investigators. A list of incident types was created, and each type was defined and numerically coded (Appendix 1). The tool included the labelling requirements outlined in the Pharmaceutical Society of Australia's *Guidelines for pharmacists providing dose administration aid services*.^7^

#### 2.1.1 Data collection procedure

A best possible medication history (BPMH) for each patient was documented by a ward pharmacist as part of routine care following admission to hospital. A BPMH is a comprehensive medication history obtained and verified using multiple information sources. Sources used included patient or carer interview, patients' own medications, community pharmacy dispensing records, residential care facility drug charts, general practitioner referral letters and My Health Record (Australia's personally controlled electronic health record). The BPMH was deemed to be the ‘gold standard’ medication history, as it was undertaken in accordance with nationally and internationally recognised procedures and included assessment of two or more sources of medication regimen information.^13^^,^^14^

An intern pharmacist or pharmacist compared the BPMH to the DAA labels and contents for each patient, then completed the data collection tool (Appendix 1). Copies of the BPMH and DAA labels were attached to the data collection tools; these were reviewed by a second investigator to ensure consistency in incident classification.

**Fig. 1:**
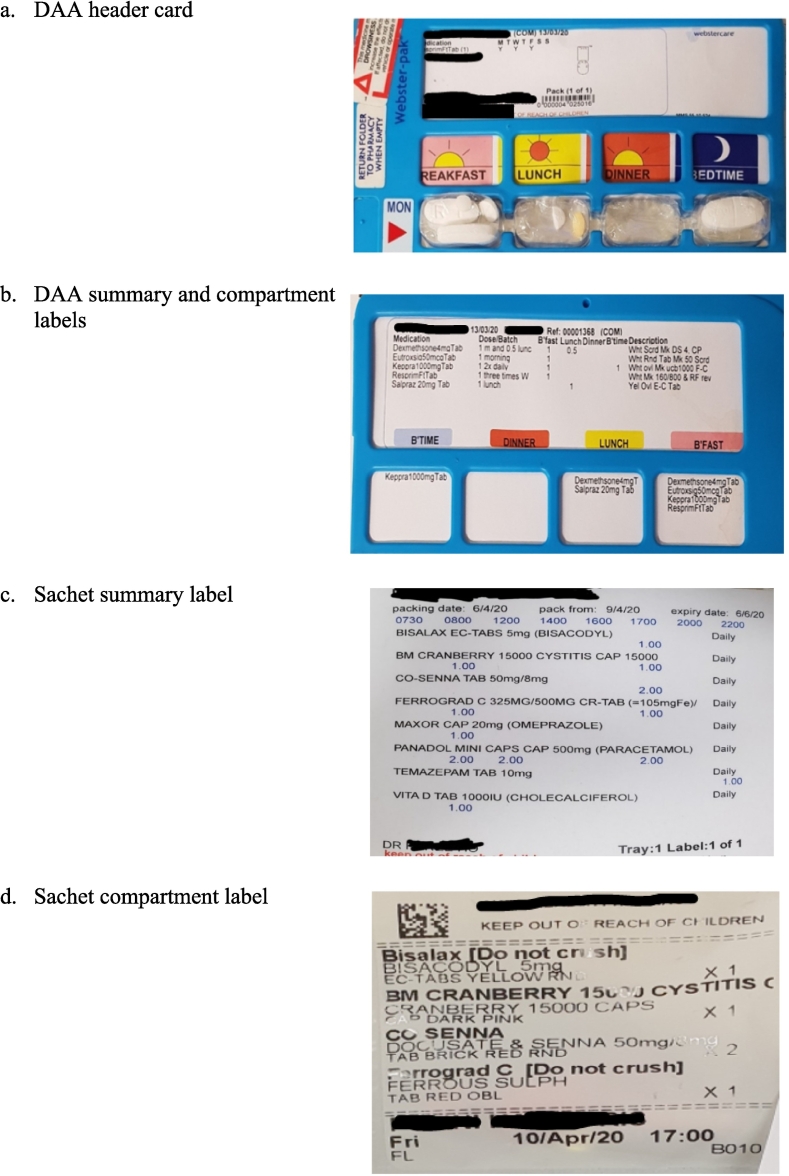
Examples of DAA header, summary and compartment labels.

Pharmacy-prepared DAAs have two places (labels) where medication information is displayed (Fig. 1), and both were reviewed:

• Header cards/Summary labels: These are usually located at the top front and/or rear of blister-pack DAAs and at the start of each roll of sachets. They should provide the patient's name, the packing pharmacy details and other data such as packing date, expiry date, storage instructions (including “keep out of reach of children”), number of DAA packs being used, whether there are non-packed medications, the patients' allergy status, and cautionary advisory labels. The summary label should provide the name, strength and form of each medication, the frequency and dose, and a description of the appearance of each medication. The Pharmaceutical Society of Australia's best-practice DAA guidelines recommend that, in addition to the brand name, the generic name should be documented.
• Individual compartment or sachet labels: These are separate labels indicating the medications packed within each individual blister or sachet for each dose-time. They may provide the name, strength, form and quantity for each medication in the particular compartment or sachet.

Evaluation of DAA labels and packed contents comprised the following steps:

1. The BPMH was compared to the DAA summary label and the DAA contents. *DAA labelling incidents* were defined as “the DAA contents match the BPMH, but the medication information on the DAA label is incorrect, missing details, illegible or ambiguous”. To be classified as “illegible or ambiguous” the information had to be presented in a manner that made it difficult to accurately ascertain the medication details (e.g. overlapping text, mis-aligned text, poor quality printing, uncommon brand name with no generic name listed, truncated dose instruction, form (capsule/tablet) and formulation (XR/IR) not being specified and strength of medication missing or truncated). *DAA packing errors* were defined as cases where the DAA contents differed from the DAA label and the BPMH, and the latter were confirmed to be correct. If during investigation of DAA-BPMH mismatches it was determined that the BPMH was incorrect, this was recorded as a *BPMH error*. This may occur, for example, when the pharmacist used an inaccurate DAA label as one of their sources for the BPMH and/or the community pharmacy reported an inaccurate medication history from an obsolete DAA profile.
2. The header cards and summary labels of each DAA (Fig. 1a,b,c,d) were reviewed for the following information specified in the Pharmaceutical Society of Australia's DAA guidelines^7^*:*
  a. Mandatory patient and packing details^7^: patient name, packing pharmacy details, packing date, expiry date (no more than eight weeks from the packing date), storage instructions, whether more than one DAA is being used, clear directions that there are more medicines in another DAA (e.g. DAA 1 of 2), “keep out of reach of children” statement and cautionary advisory label 1 (“This medicine may cause drowsiness and may increase the effects of alcohol. If affected, do not drive a motor vehicle or operate machinery”) for relevant medications.
  b. Recommended patient and packing details^7^: patient's allergy/adverse drug reaction status and whether the patient is using non-packed medications. Non-packed medications were defined as any regular medications not included in the DAA and taken in addition to the DAA (e.g. inhalers, eye drops, patches, warfarin, insulin). The BPMH was used to determine the number of non-packed regular medications for each patient.
  c. Required medication details^7^: generic name, brand name, strength, form, dose, frequency, and description of the appearance of each medication.
3. The individual compartment or sachet labels (Fig. 1b,d) were reviewed for the following medication details: name, strength and form of each medication and the number of tablets packed. Compartment labels are not a mandatory requirement in the DAA guidelines, however these were still analysed as most DAA types list the medications in each compartment.

The primary outcome was the percentage of patients whose DAA summary label(s) had one or more incidents for DAA packed medications. Secondary outcomes were: compliance with the Pharmaceutical Society of Australia's guideline for labelling DAAs; and the percentage of patients with a DAA packing error.^7^

#### 2.1.2 Data analysis

Data are reported using descriptive data analysis, including central tendency (mean or median) and variability (interquartile range [IQR]). No a priori sample size calculation was undertaken as this was a descriptive study. Microsoft Excel and Epicalc 2000 (Brixton Health, UK) were used for all analyses.

## 3 Results

### 3.1 Demographic information

One hundred and ten DAAs were reviewed for 100 patients (six patients had more than one DAA); a total of 822 medications were packed. Seventy-six patients took additional non-packed medications. Ninety-eight patients used blister-pack DAAs with 28 compartments, whilst two patients used sachet packs produced on a continuous roll. The most common brands of blister-pack DAAs were Webster Pak® (59/110, 53.6%) and Medico Pak® (29/110, 26.4%). The DAAs were prepared by 75 different pharmacies. The median patient age was 84 years and 42% were male (Table 1).

**Table 1:** Patient, medication and dose administration aid (DAA) characteristics.

*Patient characteristics (n* *=* *100)*		*95% confidence interval*
Age (years), median (IQR)	84 (73–89)	–
Sex, male, n (%)	42 (42.0)	32.3–52.3
Patient location prior to admission, n (%)		
Home	93 (93.0)	85.6–96.9
Other†	7 (7.0)	3.1–14.4
Packed medications per patient, median (IQR)	8 (6–10)	–
Non-packed medications, median (IQR)	2 (1–3)	–
		
*DAA types and brands (n* *=* *110)*‡		
Webstercare® blister pack, n (%)	62 (56.4)	46.6–65.7
Webster Pak	59 (53.6)	43.9–63.1
Flexi Pak	3 (2.7)	0.7–8.4
Medico Pak® blister pack, n (%)	29 (26.4)	18.6–35.8
Other blister-pack brand, n (%)	17 (15.5)	9.5–23.9
Sachet, n (%)	2 (1.8)	0.32–7.1

† Retirement village or nursing home.

‡ Some patients used more than one DAA.

### 3.2 Primary outcome –DAA summary label incidents

There were four/100 (4.0%) patients whose DAAs had no medication summary label. Of the 96 patients with a medication summary label on their DAA(s), 82 (85.4%) had one or more DAA summary label incidents. The most prevalent DAA summary label incidents were illegible, ambiguous, or missing medication details (75/96 [78.1%] patients had one or more such incidents), truncated medication name (27/96 [28.1%] patients) and medication omission (seven/96 [7.3%] patients) (Table 2). Descriptions of the incident types and examples are outlined in Table 3.

**Table 2:** Dose administration aid (DAA) summary label incidents.

Types of DAA summary label incidents†	Number of patients with ≥1 incident, n (%)	95% confidence interval	Median number of incidents per patient	Range of incidents per patient
Illegible, ambiguous or missing medication details	75 (75.0)	65.2–82.9	2	0–13
Truncation of medication name	27 (27.0)	18.8–37.0	0	0–5
Omission	7 (7.0)	3.1–14.4	0	0–2
Incorrect dose	6 (6.0)	2.5–13.1	0	0–1
Incorrect frequency or dose time	6 (6.0)	2.5–13.1	0	0–1
Missing dosage and administration directions	5 (5.0)	1.86–11.83	0	0–2
Commission	3 (3.0)	0.8–9.2	0	0–1
Incorrect dose-form	2 (2.0)	0.35–7.7	0	0–1

† DAA summary label incident = The medication is correctly packed (i.e. the best possible medication history and DAA contents match), but the medication information on the DAA label are incorrect, missing details, illegible or ambiguous.

**Table 3:** Dose administration aid (DAA) summary label incident types.

Types of DAA label incident	Incident description	Examples from audited DAAs
Illegible, ambiguous or missing medication details	• Illegible instructions (e.g. overlapping text) • Ambiguous medication name • Missing medication details: strength (e.g. 20 mg), units (e.g. mg, mcg), formulation (e.g. extended release [XR], enteric coated, etc.], dose-form (tablets, capsules) • Ambiguous dose regimen	• Illegible name for both fluvoxamine and ranitidine as the “supper” heading was covering it. • “Rithmik” brand name used for amiodarone without accompanying generic name (uncommon brand name, potentially ambiguous for hospital prescriber) • Strength of furosemide missing • Perindopril/indapamide listed on DAA label but dose units not specified • Metoprolol XR was packed, but not labelled as “XR”. • Potassium slow release was packed alternate daily. The instruction on the label stated “one morning every” and the words “alternate day” were cut off.
Truncation of medication name	• Medication name truncated on label	• Sertraline 50 mg truncated to “S 50 mg”
Omission	• Medication name omitted from the label despite medication being correctly packed in DAA	• Metoprolol and telmisartan were not on the DAA summary label or the compartment labels, but were correctly packed.
Missing dosage or administration directions	• Number of tablets or administration directions, including time and frequency, not specified	• Folic acid dosage directions missing on summary label
Incorrect dose	• Different number of tablets/capsules from what is correctly packed	• Metoprolol dose incorrectly documented on the DAA label as one tablet twice a day when it was correctly packed as a quarter of a tablet twice a day.
Incorrect frequency or dose time	• Different frequency/dose-time from what is correctly packed	• Madopar® (levodopa-benserazide) was correctly packed as two tablets in the morning, one tablet at 9 am, one tablet at 1 pm, however the label had the directions as one tablet twice a day with no specific times stated.
Commission	• Medication listed as packed on DAA label but correctly it is correctly not in the pack	• Donepezil was listed on the DAA summary label but correctly not packed in the DAA because it had been ceased one month earlier. • Levothyroxine was on the DAA label but it was correctly not packed as the patient was taking it outside of the pack.
Incorrect dose-form	• Dose form specified on the label is different from what is correctly packed	• Mirtazapine oral disintegrating tablet (ODT) listed on the summary label, but the plain tablets were correctly packed in the DAA.

### 3.3 Other DAA incidents

There were 13/110 (11.8%) DAAs (from 12/100 patients) with no individual compartment content labelling (all were blister packs). There were 12 other DAA-related incidents identified: no summary label (*n* = 8 DAAs from four patients), failure to seal the DAA resulting in medicines being mixed between the compartments (*n* = 1 DAA) and the medication brand packed did not match with the brand (and thus the tablet/capsule description) on the DAA label (*n* = 3 DAAs). Two packing errors (for two patients) were identified; one was an incorrect dose form and the other was an omission. Four BPMH errors were identified (ceased, missing or incorrect dosing instructions documented by the ward pharmacist).

### 3.4 Compliance with best-practice DAA labelling guidelines

**Fig. 2:**
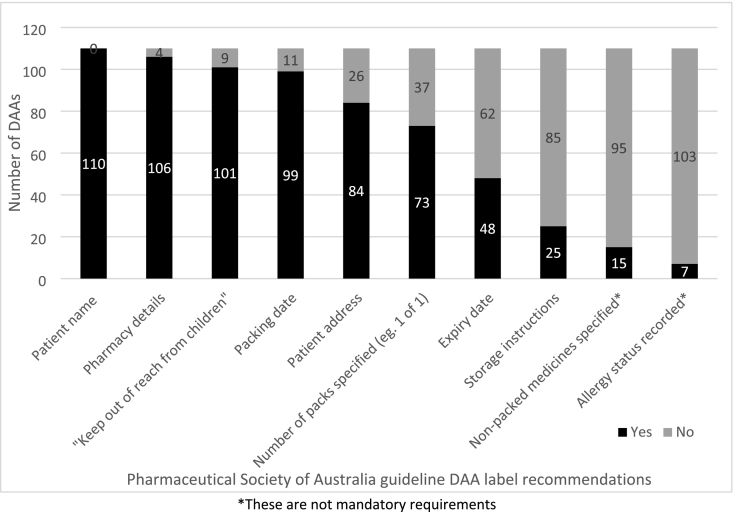
Inclusion of required and recommended components on the header card/summary label for pharmacy-prepared dose administration aids (DAAs) (*n* = 110).

DAA header cards and/or summary labels lacked the following patient and packing details: storage instructions (85/110, 77%), expiry date (62/110, 56%), number of DAA packs (37/110, 34%), patient address (26/110, 24%) and packing date (11/110, 10%) (Fig. 2).

Of the 48 DAAs with an expiry date on the label, 21 had an expiration duration of more than the guideline-recommended maximum of eight weeks.^7^ Of the 49 DAAs that required a cautionary advisory label 1 (drowsiness warning), 15 (30.6%) did not have one (Fig. 2).

Of the 822 medications listed on DAA summary labels, 561 (68%) were not documented with their generic name and 119 (14.5%) did not have a description of the appearance on the label to facilitate identification in the pack.

Forty-three patients had a known medication allergy/ADR, but only one (2.3%) had allergy/ADR status documented on the DAA label and it was inaccurate. It said no “known drug allergy” but the patient had allergies to sulfamethoxazole-trimethoprim and sulfasalazine. Of the 57 patients who did not have a known allergy/ADR, only six (10.5%) patients had “no known drug allergy” or similar wording documented on their DAA label.

Only 15/110 of the DAA labels had documentation regarding whether the patient used non-packed medications. Of these 15, three (20.0%) correctly listed what the non-packed medications were, four (26.7%) provided an incomplete list of the patient's non-packed medications and eight (53.3%) stated there were no non-packed medications when the patient was taking medications in addition to those in the DAA.

#### 3.4.1 Other DAA labelling observations

Some Webster Pak® branded DAAs had a plastic frame that encased the blister pack to provide integrity to the pack. Of the 59 Webster Pak® branded DAAs in this study, 36% had DAA medication summary and/or compartment label text that was covered by the plastic frame, making it difficult to read the contents of the pack. These were not counted as incidents (the frames were opened to review information covered by the plastic case.)

**Fig. 3:**
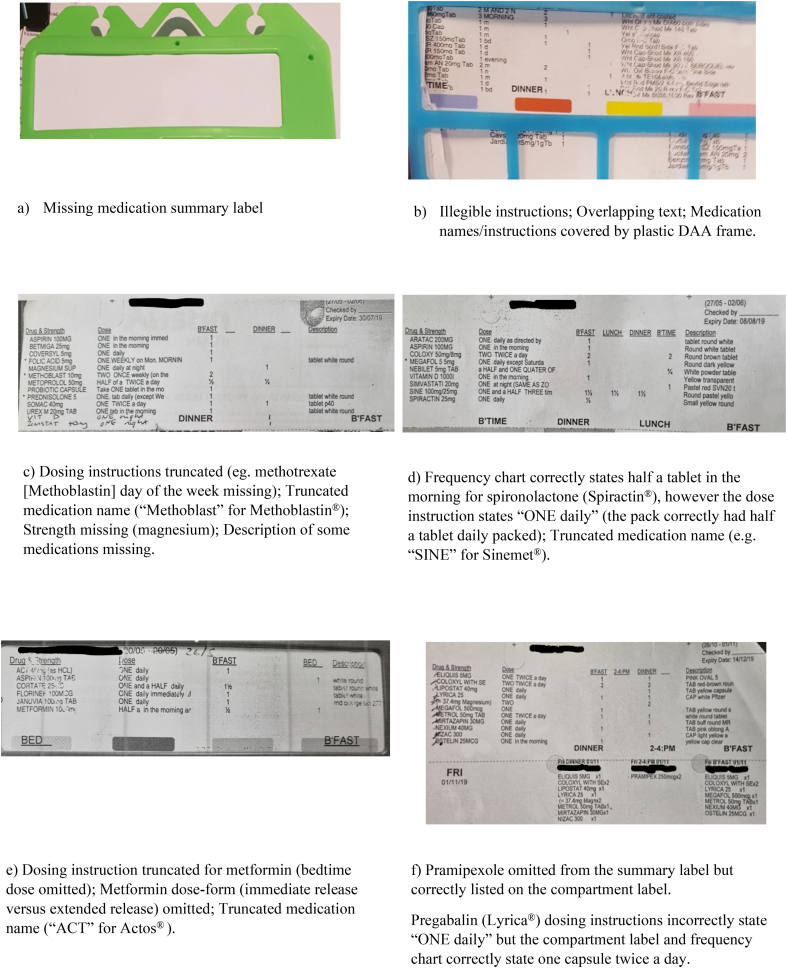
Examples of DAA summary labels evaluated.

Examples of audited DAA packs and common labelling issues are provided in Fig. 3.

## 4 Discussion

In this study the majority of labels on pharmacy-prepared DAAs contained errors or lacked clarity. Four percent of patients' DAAs had no medication summary label. When patients' DAAs did have a label, 85% had one or more summary label incidents related to DAA-packed medications. Best-practice DAA labelling guidelines were poorly adhered to. The prevalence of DAA packing errors was low.

There is limited research on the accuracy and clarity of DAA labelling. Most previous studies evaluating issues with DAAs were undertaken in nursing homes^9^^,^^11^^,^^15^, ^16^, ^17^ and focused on discrepancies between DAA contents and nursing home medication charts, along with other DAA packing incidents such as damaged medications, inappropriately altered/divided medications and medications unsuitable for re-packing according to pharmaceutical guidelines.^11^^,^^15^, ^16^, ^17^ This study focussed mainly on the quality of DAA labelling because it is the label that is used by hospital medical staff when determining the admission medication history; it would be rare for medical staff to also check the contents of the DAA.

Three previous studies have reported on the accuracy of DAA labels.^9^, ^10^, ^11^ An Australian study by Chen, et al. evaluated the accuracy of various information sources available to emergency department clinicians, to assess their utility for obtaining a BPMH.^10^ They found that where a DAA was brought to the emergency department, there was a discrepancy between the BPMH and the DAA label for 88.6% patients. These findings by Chen, et al. are not comparable to this study because they included omission of non-packed medications from the DAA label as an incident, whereas the primary outcome of this study focused on packed medications. Also, their study included both pharmacy-prepared and patient/carer-prepared DAAs. In addition, they did not evaluate label ambiguities and compliance with best-practice labelling requirements.^7^ Another Australian study compared DAA contents to medication charts for 2480 residents at 42 nursing homes.^11^ They reported incorrect labels for seven patients, but the method for assessing labels and the nature of these errors were not reported.^11^ A UK study evaluated medication prescribing, dispensing and administration errors at 55 nursing homes (256 patients, 86% of whom had medications packed in a DAA).^9^ Labelling errors were found for 7.3% of dispensed items, and content errors for 2.3%. Details regarding the nature of these errors was not reported.^9^

This study detected only two packing errors (0.24% of 822 packed medications), for two/100 (2.0%) patients. Other studies have reported higher rates of inaccurate DAA content, affecting up to 11.5% of DAAs.^9^^,^^11^^,^^15^, ^16^, ^17^ This difference may be explained by different medication incident/error definitions, study populations and methodologies. The most common incidents in previous studies were damaged medications, inappropriately altered/divided medications, and medications unsuitable for re-packing,^11^^,^^15^, ^16^, ^17^ but this study did not include these incidents as they are not related to accuracy of DAA packing. Previous studies were undertaken in the nursing home setting, whereas over 90% of patients in this study were residing in the community before they presented to hospital. There are differences in the workflow for prescribing medications and packing DAAs for community versus nursing home residents. Previous studies compared DAA contents with nursing home medication charts. Discrepancies between nursing home medication charts, doctors' prescriptions and pharmacy dispensing profiles are known to occur,^18^ so differences between DAA contents and nursing home charts may reflect problems with communication of information between prescribers, pharmacies and nursing homes, rather than DAA packing errors by pharmacies.^19^ Carruthers, et al. reported that a substantial number of DAA errors were associated with failure to provide prescriptions or failure to chart or communicate medication changes to the pharmacy.^11^ Hussainy, et al. stated that numerous errors may have occurred due to a lack of effective communication between the nursing homes and pharmacy.^16^ Barber, et al. reported that prescriptions and medication administration records (charts) were often different, so it was unclear which was correct.^9^

Consistent with this study, Carruthers et al.^11^ also noted that most medications were labelled only by brand name. They reported that the use of brand names on DAA labels caused confusion for nurses, particularly if the prescriber charted the medication by a different name. Similarly, this study identified that 68% of the medications were listed by brand name alone. Listing the brand name alone at times makes it unclear for doctors and nurses what the active ingredient is, particularly if it is an uncommon brand name. DAA labels frequently did not include a product description for packed medications. The generic name and description can be useful details for patients, nurses and carers to identify medications within DAAs.

Patients who use DAAs are often unable to describe their medication regimen in detail.^3^^,^^8^ Therefore, the DAA label is a valuable tool for prescribers and other health professionals to facilitate prescribing and therapeutic decision-making as patients move from one setting of care to another. Australian hospital pharmacists have reported that incomplete DAA profiles resulted in confusion regarding patients' medication regimens when they are admitted to hospital.^20^ A major pharmacy indemnity insurer in Australia recently reported an increase in claims relating to DAA incidents.^20^^,^^21^ One of the reasons was reported to be pharmacy staff packing medications based on an obsolete medication profile. The results from this study are consistent with these reports. There were cases where medication regimen changes were handwritten on the DAA label by community pharmacy staff. Handwritten changes may increase the risk of having obsolete medication profiles stored in DAA labelling programs. Hence, pharmacies providing DAA services need to ensure they have robust procedures in place to ensure up-to-date medication profiles are maintained.

Most DAA labels that were evaluated in this study lacked appropriate expiry dates and storage instructions. There is limited data on the stability of medications outside of their original packaging.^22^^,^^23^ The guidelines published by peak pharmacy organisations internationally have variable recommendations regarding the expiry of repackaged medications.^22^ The expiration dates range from 28 days (Denmark), eight weeks (United Kingdom) and up to 60 days (United States of America).^22^ Australian guidelines recommend an eight week expiry.^7^ In this study, only 44% of DAAs had an expiry date recorded on the label, and when it was recorded, it was more than eight weeks for almost half of the DAAs. Given that some medications have specific storage directions and stability profiles, DAA labelling software vendors need to ensure that there is a field for pharmacists to insert an appropriate expiry date or at least standardise the expiration duration to a maximum of eight weeks.

Details such as patient allergy status and medications taken outside of the DAA are non-mandatory details which can be provided on DAA labels.^7^ Most DAA labels did not include allergy status and non-packed medications. When these details were documented, they were mostly inaccurate or incomplete. For example, a DAA label incorrectly documented ‘no known drug allergies’, but the patient had an allergy to sulfamethoxazole/trimethoprim and sulfasalazine. If packing pharmacies choose to provide additional information such as allergy status, it is imperative that they ensure that it is regularly updated.

Several software related labelling incidents were identified, including medication names being truncated, and the medication name, strength and/or unit missing due to the lack of space on the label. There were incidents where information on the label was covered by ancillary labels and by the plastic framework that came with certain DAA packaging. This is consistent with findings from the UK study, where Barber et al. reported that factors associated with DAA label errors were the computer systems used and that some DAAs did not have space to fit all the required labels.^9^ Vendors of DAA label software need to ensure that their labelling programs are able to provide labels that meet current guidelines for patient safety. DAA labelling programs need to standardise the information that goes onto a DAA label. If it is impossible for the DAA software to accommodate these labelling requirements due to the limited space, current guidelines need to be reviewed and adjusted to prioritise the information to be included on the DAA label. In addition, pharmacy staff packing DAAs need to take greater care when producing DAA labels to ensure that the labels accurately and clearly reflect the contents within DAA pack.

### 4.1 Strengths and limitations

Strengths of this study were inclusion of DAAs from a broad range of pharmacies, comparison of DAAs with a verified medication history (BPMH) and thorough review of both the label and contents of each DAA, with a comprehensive definition of packing and labelling errors. The study analysed DAAs from 75 different pharmacies which supports the generalisability of the results. The study did however have several limitations. Given the small sample size, it was not possible to make comparisons between the various DAA brands and software. Patients were selected through convenience sampling which has the potential to introduce selection bias. To minimise bias, a decision to include a DAA in the study was undertaken prior to reviewing the quality and accuracy of the DAA. DAAs from all areas of the hospital were included, therefore patients in hospital for acute, subacute and rehabilitation care were included. Whilst patients were recruited using snap-shot audits over three years, no new DAA guidelines were introduced over the study period. This study did not evaluate whether labelling errors translated to prescribing errors or adverse outcomes, as ward pharmacists at the study hospital routinely intervene to correct orders when doctors chart medications on admission. Despite the ‘gold standard’ pharmacist documented BPMH being undertaken in accordance with best-practice, four BPMH errors were identified (for four patients). For example, there was an incident where donepezil was not packed in the DAA, and this was correct because it was stopped by the prescriber one month earlier. However, it was still listed on the DAA summary label and was also listed on the BPMH; this incident was both a DAA label and a BPMH error. The BPMH error likely occurred because the DAA summary label was incorrect, and was a result of obsolete medication profiles saved on DAA software programs being communicated to ward pharmacists creating the BPMH. Packing errors were a secondary endpoint in this study. There is the potential that this study may have under-estimated packing errors as most DAAs evaluated were partially-used or in some cases empty at the time of hospital admission, so it was not possible to check the contents of an entire one weeks' worth of packing for accuracy.

## 5 Conclusion

Most pharmacy-prepared DAA labels contained one or more incidents and were inconsistent with best-practice guidelines. The prevalence of packing errors was low. These findings suggest that the providers of DAA labelling software need to improve the functionality of their software to facilitate clarity. Pharmacies providing DAA services need to develop more robust procedures and educate their staff involved in preparing DAAs about the use of DAA software, to minimise labelling errors. Improved checking processes during DAA packing are recommended to ensure that labels are accurate, clear and comprehensive. Best-practice guidelines should rationalise the information required on the DAA label or nominate the labelling requirements that are essential and those that are recommended only if label space permits without compromising clarity.

## Declaration of Competing Interest

None.

## Figures and Tables

**Figure:**
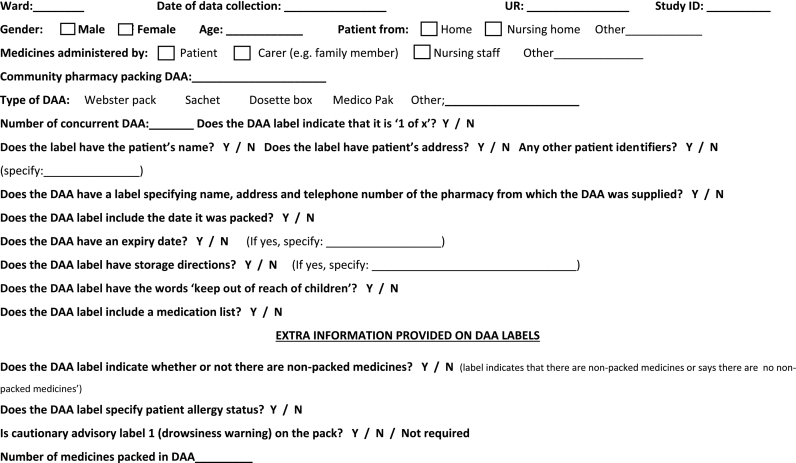


**Figure:**
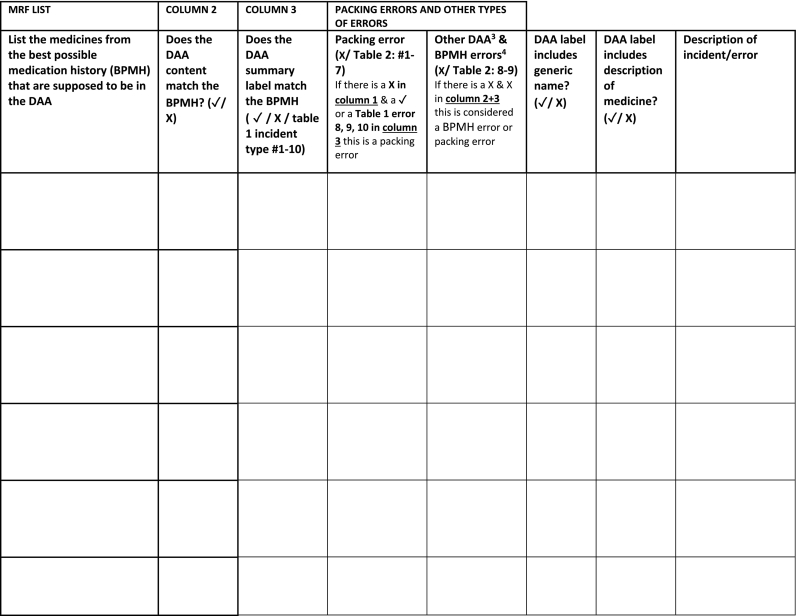


**Figure:**
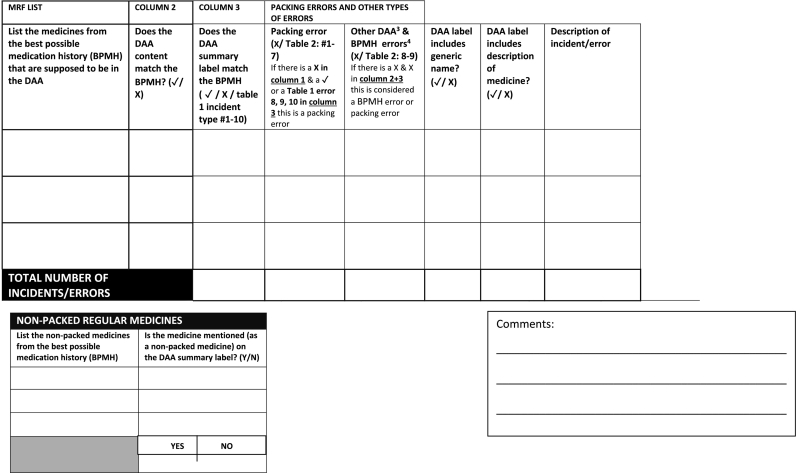


**Figure:**